# A Salt Bridge Linking the First Intracellular Loop with the C Terminus Facilitates the Folding of the Serotonin Transporter*

**DOI:** 10.1074/jbc.M115.641357

**Published:** 2015-04-13

**Authors:** Florian Koban, Ali El-Kasaby, Cornelia Häusler, Thomas Stockner, Benedikt M. Simbrunner, Harald H. Sitte, Michael Freissmuth, Sonja Sucic

**Affiliations:** From the ‡ Institute of Pharmacology, Center of Physiology and Pharmacology, Medical University of Vienna, A-1090 Vienna, Austria and; the § Department of Pharmacology, Faculty of Veterinary Medicine, Mansoura University, 35516 Mansoura, Egypt

**Keywords:** dopamine transporter, endoplasmic reticulum (ER), protein folding, serotonin transporter, trafficking, amphipathic helix, pharmacochaperoning

## Abstract

The folding trajectory of solute carrier 6 (SLC6) family members is of interest because point mutations result in misfolding and thus cause clinically relevant phenotypes in people. Here we examined the contribution of the C terminus in supporting folding of the serotonin transporter (SERT; SLC6A4). Our working hypothesis posited that the amphipathic nature of the C-terminal α-helix (Thr^603^–Thr^613^) was important for folding of SERT. Accordingly, we disrupted the hydrophobic moment of the α-helix by replacing Phe^604^, Ile^608^, or Ile^612^ by Gln. The bulk of the resulting mutants SERT-F604Q, SERT-I608Q, and SERT-I612Q were retained in the endoplasmic reticulum, but their residual delivery to the cell surface still depended on SEC24C. This indicates that the amphipathic nature of the C-terminal α-helix was dispensable to endoplasmic reticulum export. The folding trajectory of SERT is thought to proceed through the inward facing conformation. Consistent with this conjecture, cell surface expression of the misfolded mutants was restored by (i) introducing second site suppressor mutations, which trap SERT in the inward facing state, or (ii) by the pharmacochaperone noribogaine, which binds to the inward facing conformation. Finally, mutation of Glu^615^ at the end of the C-terminal α-helix to Lys reduced surface expression of SERT-E615K. A charge reversal mutation in intracellular loop 1 restored surface expression of SERT-R152E/E615K to wild type levels. These observations support a mechanistic model where the C-terminal amphipathic helix is stabilized by an intramolecular salt bridge between residues Glu^615^ and Arg^152^. This interaction acts as a pivot in the conformational search associated with folding of SERT.

## Teaser

**Background:** The C terminus of the serotonin transporter (SERT) is required for folding.

**Results:** Replacing C-terminal residues (Phe^604^, Ile^608^, and Ile^612^ by Gln and Glu^615^ by Lys) caused misfolding of SERT. A charge reversal (R152E) rescued SERT-E615K.

**Conclusion:** An amphipathic C-terminal helix interacts with the first intracellular loop to facilitate folding of SERT.

**Significance:** These data provide insights into the folding trajectory of SERT and related transporters.

## Introduction

The serotonin transporter (SERT)^3^ belongs to the monoamine transporter subfamily of the solute carrier 6 (SLC6) protein superfamily. It is a membrane protein comprising a cytosolic N terminus of 85 residues, 12 transmembrane helices, and a cytosolic C terminus of 35 residues. SERT is predominantly expressed on the presynaptic terminals of raphe neurons. Its currently understood physiological role is to retrieve serotonin from the synaptic cleft into the presynaptic compartment. This action is crucial for both terminating and sustaining serotoninergic signaling. Like all membrane proteins, SERT is translated into the ER. Export from the endoplasmic reticulum is exclusively supported by the SEC24 isoform SEC24C (1, 2). Delivery of SERT to the axonal compartment and hence to the presynaptic specialization is also contingent on SEC24C (3). Mutations in the C-tail of SERT lead to misfolding and ER retention of the transporter (4). The folding defects can be remedied in part by ibogaine (4) and by its more potent analog noribogaine (5). Ibogaine binds to and hence stabilizes the inward facing conformation (6, 7); in contrast, ligands that bind to the outward facing conformation fail to rescue folding-deficient SERT mutants (4). This indicates that the folding trajectory of SERT proceeds through the inward facing conformation (4, 8).

Folding of SERT is contingent on the recruitment of heat shock proteins (HSPs) to the C terminus of SERT; individual folding-deficient mutants of SERT are trapped in complexes that differ in their composition of HSPs and associated co-chaperones. This indicates that a relay of HSPs operates on folding intermediates of SERT (5). Accordingly, some but not all folding-deficient mutants of SERT can be rescued by inhibiting HSP70 and/or HSP90 (5). Single point mutations in the coding sequences of SLC6 family members result in misfolding of the cognate protein and thus give rise to folding diseases (8). Prominent examples of these include mutants of the dopamine transporter (DAT; SLC6A3) (9–11) and of glycine transporter 2 (SLC6A5) (12–14). By analogy with SERT mutants, it is very plausible that these naturally occurring mutants of DAT and glycine transporter 2 may also be rescued by pharmacochaperoning and/or manipulation of HSP isoform levels. In addition, it is evident that attempts to rescue folding-defective mutants should benefit from insights into the folding trajectory of SLC6 transporters. A model has been proposed where a relay of cytosolic heat shock proteins are recruited to the C terminus of SLC6 transporters to shield the SEC24-binding site (8). This HSP/COPII exchange model posits that the folding intermediates are relayed between individual HSPs until a stable conformation is reached. This precludes premature recruitment of the COPII coat and thus export of incompletely folded transporters from the ER (5, 8). The HSP/COPII exchange model also implies that the C terminus participates in the conformational search involved in adopting the folded state. Hence, in the present study, we explored how folding of SERT is supported by its C terminus. Our observations show that (i) the amphipathic nature of a C-terminal α-helix is essential for folding but not for ER export of SERT and that (ii) conformational search is assisted by a salt bridge between the C-terminal α-helix bridge and the first intracellular loop (IL-1), *i.e.* via an interaction of Glu^615^ and Arg^152^.

## Experimental Procedures

### 

#### 

##### Materials

[^3^H]5-Hydroxytryptamine ([^3^H]5-HT; serotonin; 28 Ci/mmol) and [^3^H]imipramine (48 Ci/mmol) were purchased from PerkinElmer Life Sciences. Cell culture media, supplements, and antibiotics were from Invitrogen. Noribogaine was a generous gift of Sacrament of Transition (Maribor, Slovenia). Bovine serum albumin (BSA) and Complete^TM^ protease inhibitor mixture were from Roche Applied Science, SDS was from BioMol GmbH (Hamburg, Germany), and Tris and scintillation mixture (Rotiszint® eco plus) were from Carl Roth GmbH (Karlsruhe, Germany). Anti-GFP antibody (ab290) was from Abcam Plc (Cambridge, UK). Protein A-Sepharose and anti-rabbit IgG1 antibody linked to horseradish peroxidase were from Amersham Biosciences. All other chemicals were of analytical grade. Predesigned Stealth RNA duplex oligoribonucleotides used to deplete SEC24C were purchased from Invitrogen as described previously (1) along with the appropriate negative controls (Invitrogen). The 0.4% trypan blue stock solution was purchased from Sigma-Aldrich. Antiserum 7 was raised against amino acids 8–23 of the G protein β_1_-subunit and recognizes Gβ_1_ and Gβ_2_ (15). Immunostaining for Gβ subunits was used as a loading control.

##### Mutagenesis, Cell Culture, and Transfection

Mutations were introduced into plasmids encoding CFP-SERT or YFP-SERT using the QuikChange Lightning site-directed mutagenesis kit (Agilent Technologies, Santa Clara, CA). The mutagenic primers were designed using the QuikChange primer design tool provided by the manufacturer. HEK93 cells were grown at 37 °C in a 5% CO_2_ humidified atmosphere in Dulbecco's modified Eagle's medium (DMEM) supplemented with 10% fetal calf serum, 60 mg/liter penicillin, and 100 mg/liter streptomycin. CAD cells were grown in Ham's F-12/DMEM (1:1) medium supplemented with 8% fetal calf serum, 60 mg/liter penicillin, and 100 mg/liter streptomycin. Cell differentiation was induced by serum removal for 24 h as described previously (1). The cells were transfected using Lipofectamine 2000 or Lipofectamine^TM^ reagent combined with the Plus^TM^ reagent (all from Life Technologies) according to the protocols provided with the reagents. HEK293 cells were transfected with Stealth RNA duplex oligoribonucleotides and the appropriate negative controls (Invitrogen) using Lipofectamine RNAiMAX (Invitrogen). After 48 h, the cells were transfected with plasmids (0.5–2 μg/10^6^ cells) encoding the transporters using Lipofectamine 2000.

##### Radiotracer Assays and Confocal Microscopy

[^3^H]5-HT uptake and [^3^H]imipramine binding were performed as described previously (4). In brief, HEK293 cells were transfected with the indicated SERT constructs. For uptake assays, transfected cells were seeded onto poly-d-lysine-coated 48-well plates to a density ∼10^5^ cells/well. After 24 h, the cells were washed with Krebs-HEPES buffer (10 mm HEPES, 120 mm NaCl, 3 mm KCl, 2 mm CaCl_2_, 2 mm MgCl_2_, 2 mm glucose monohydrate, pH 7.3) and incubated for 1 min with 0.2 μm [^3^H]5-HT. For experiments where the *K_m_* and *V*_max_ were determined, the specific activity of [^3^H]5-HT was diluted with unlabeled 5-HT to achieve final concentrations of 0.2, 1, 3, 10, 1, and 30 μm. Background uptake was determined in the presence of the specific SERT inhibitor paroxetine (10 μm). For [^3^H]imipramine binding, transfected cells were mechanically scraped into PBS and collected by centrifugation. Cellular membranes were prepared by a combination of hypotonic lysis (25 mm HEPES, 2 mm MgCl_2_, 1 mm EDTA, pH 7.3), freeze/thaw cycles using liquid N_2_, and sonication. Membranes were collected by centrifugation and resuspended in assay buffer (20 mm Tris·HCl, pH 7.5, 1 mm EDTA, 2 mm MgCl_2_, 120 mm NaCl, 3 mm KCl). Membranes (16–20 μg/assay) were incubated with the indicated concentration of [^3^H]imipramine at room temperature for 20 min. Reactions were terminated by trapping membranes on glass fiber filters precoated with polyethyleneimine. After rapid washing with buffer, filters were dissolved in scintillation mixture, and radioactivity was measured by liquid scintillation counting.

For imaging, HEK293 or CAD cells expressing wild type YFP- or CFP-tagged SERT or SERT mutants were seeded onto poly-d-lysine-coated ibidi® glass bottom chambers. After 24 h, cell imaging was performed. Confocal microscopy was done using a Zeiss LSM510 microscope equipped with an argon laser (at 30 milliwatts) and a 63× oil immersion objective (Zeiss Plan-Neofluar). At least 10 individual cells per condition were analyzed by ImageJ using the built-in plot profile analysis to quantify the expression pattern of individual mutants in the absence or presence of the pharmacochaperone noribogaine. The plasma membrane was delineated by staining with trypan blue. Alternatively, a plasmid driving the expression of myristoylated and palmitoylated CFP was co-transfected with YFP-tagged versions of SERT because trypan blue and YFP emission spectra overlap.

##### Cell Lysis, SDS-PAGE, Immunoblotting, and Cell Surface Biotinylation

Adherent SERT-expressing cells were washed with and scraped into PBS. Cells were collected by centrifugation and membranes were solubilized in lysis buffer (50 mm Tris·HCl, pH 8.0, 150 mm NaCl, 1% DDM, 1 mm EDTA, protease inhibitor mixture). Detergent-insoluble material was removed by centrifugation (16,000 × *g*, 15 min, 4 °C). Proteins in the supernatant were denatured in SDS-PAGE sample buffer, and aliquots were resolved by SDS-PAGE. Proteins were transferred onto nitrocellulose membranes, which were blocked with 5% bovine serum albumin in 0.1% TBS, Tween 20. Membranes were incubated with anti-GFP antibody (ab290) at a 1:3000 dilution in TBS, Tween 20 overnight at 4 °C. Subsequently, membranes were washed in TBS, Tween 20; horseradish peroxidase (HRP)-conjugated secondary antibody (1:5000) was applied; and the immunoreactivity was detected by chemiluminescence. Biotinylation of cell surface proteins was done as described previously (2). Briefly, CAD cells were incubated in the presence of sulfo-NHS-SS-biotin (sulfosuccinimidyl-2-(biotinamido)ethyl-1,3- dithiopropionate; Pierce; 1 mg/ml) for 30 min. After cell lysis, the labeled surface proteins were retrieved by binding to streptavidin beads (Thermo Fischer Scientific Inc.). The amount of biotinylated SERT was quantified by immunoblotting.

##### Molecular Modeling

A sequence alignment between *Drosophila melanogaster* DAT (dDAT) and the human SERT was created using MUSCLE (16) as published previously (17). The structure of dDAT (Protein Data Bank code 4M48 (18)) was used as the reference template for creating the homology model of the human SERT transporter. The EL2 loop of the dDAT template differs in sequence from the human SERT; it is truncated and strongly interacts with the co-crystallized antibody. We therefore used the EL2 loop from our best model of human DAT (17) as a template for the EL2 loop. The complete C terminus was added and modeled as a loop structure because structure predictions indicated that the C terminus should be largely unstructured after the helix identified in the crystal structure of the dDAT. MODELLER version 9.12 (19) was used to create 100 structures using the automodel procedure. Model quality was evaluated using the DOPE score (20). The four best models were inserted into a system consisting of a pre-equilibrated membrane created to harbor the SERT using the g_membed method (21). After equilibration of the surrounding environment for 2.5 ns, position restraints on SERT were slowly reduced in four steps, applying 1000, 100, 10, and 1 kJ/mol, respectively, each time simulating for 2.5 ns. Production simulations were carried out for 100 ns. Molecular dynamics simulations were carried out using the GROMACS 4.6.3 MD package (22), applying the OPLS force field (23). The 1-palmitoyl-2-oleoyl-*sn*-glycero-3-phosphatidylcholine lipids of the membrane were represented by Berger lipids (24) converted into the format of the OPLS all-atom force field by following a proposed procedure.^4^ The water was represented as simple point charge water. Bonds were constrained using LINCS (25). The simulations were carried out at a constant temperature of 310 K using the v-rescale (τ = 0.1 ps) thermostat (26), coupling the protein, membrane, and water/ions separately. The pressure was maintained at 1 bar applying the weak coupling algorithm (27) with a coupling constant of 1.0 ps and a compressibility of 4.5 × 10^−5^ bar^−1^. The electrostatic interactions were evaluated using the smooth particle mesh Ewald method (28) with a cutoff of 1.0 nm. The Lennard-Jones interactions were evaluated using a cutoff of 1.0 nm. Long range correction for energy and pressure was applied.

## Results

### 

#### 

##### Mutations within the Hydrophobic Segment of the Amphipathic Helix Impair Folding and Surface Expression

**FIGURE 1.**
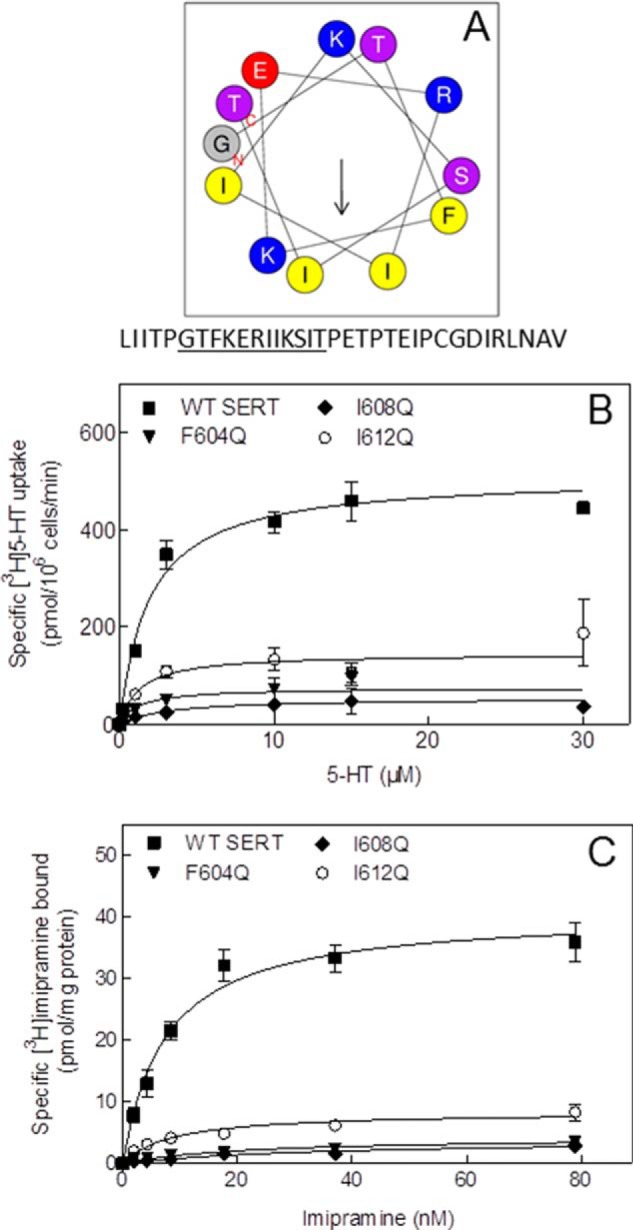
**SERT requires a C-terminal amphipathic helix for correct surface expression.** *A*, the C terminus of SERT contains an amphipathic helix. The *arrow* represents the hydrophobic moment of the helix. The amino acid sequence between Gly^602^ and Thr^613^ was plotted using a computational helical wheel projection tool (HeliQuest). This results in an amphipathic α-helix in which the hydrophobic residues Phe^604^, Ile^608^, and Ile^612^ are located on one side of the helix, and polar and charged amino acids are located on the other side. Mutations on the hydrophobic side of the helix lead to reduced substrate uptake (*B*) and reduced ligand engagement (*C*). Uptake of [^3^H]5-HT and binding of [^3^H]imipramine were determined in three independent experiments as described under “Experimental Procedures.” *Error bars* represent S.E.

Recently, a crystal became available showing the structure of *D. melanogaster* DAT (18). One feature of this structure is a helix of 2.5 turns between the residues Leu^586^ and Thr^595^. We aligned the *Drosophila* DAT amino acid sequence with that of the human SERT (not shown). Consistent with a recent structural prediction (29), we found that the corresponding region (Phe^604^–Thr^613^) in human SERT fulfills the requirements for an α-helix at this position according to the theoretical propensity of different amino acids to participate in an α-helix (30, 31). Within this region, the C terminus of SERT contains four hydrophobic residues (*i.e.* Phe^604^, Ile^608^, Ile^609^, and Ile^612^), three of which are within distances of four amino acids to the previous one (*i.e.* Phe^604^, Ile^608^, and Ile^612^). All amino acids in this stretch are suitable for creating an α-helix, and one turn of an α-helix contains 3.6 amino acids per turn. Hence, we hypothesized an amphipathic α-helix in which hydrophobic amino acids are located on one side of the helix and hydrophilic amino acids on the other. This was confirmed by plotting the appropriate sequence using a helical wheel projection program (HeliQuest) (Fig. 1*A*).

Alanine substitutions in the positions 604, 608, and 612 are tolerated and do not impair folding of SERT (4). However, if the amphipathic nature of the helix was of significance, disruption of the hydrophobic moment ought to have phenotypic consequences. Accordingly, we examined the consequences of introducing glutamine mutations at these positions. YFP-tagged SERT-F604Q, SERT-I608Q, and SERT-I612Q were transiently expressed in HEK293 cells, and the uptake of [^3^H]5-HT was determined; maximum velocity of transport (*V*_max_) is related to the number of transporters at the cell surface. This value was reduced (see Fig. 1*B*; *V*_max_ = 510 ± 25, 75 ± 11, 54 ± 25, and 145 ± 14 pmol × 10^−6^ cells × min^−1^ for wild type SERT, SERT-F604Q, SERT-I608Q, and SERT-I612Q, respectively). In contrast, *K_m_* values were comparable, *i.e.* 1.9 ± 0.4, 1.3 ± 0.8, 3.0 ± 0.4, and 1.3 ± 0.5 μm for wild type SERT, SERT-F604Q, SERT-I608Q, and SERT-I612Q, respectively. This indicates that substrate affinity was not affected. We also verified that the turnover number was not altered by quantifying binding-competent transporters with the radioligand [^3^H]imipramine (Fig. 1*C*): the level of wild type SERT and the SERT mutants assessed by binding (*B*_max_ = 41 ± 2.2, 4.3 ± 0.7, 3.9 ± 0.9, and 8.4 ± 0.7 pmol/mg for wild type SERT, SERT-F604Q, SERT-I608Q, and SERT-I612Q, respectively) paralleled that measured by substrate uptake; thus, the turnover rate, *i.e.* the ratio of *V*_max_/*B*_max_ was similar (129, 124, 128, and 164 min^−1^ for wild type SERT, SERT-F604Q, SERT-I608Q, and SERT-I612Q, respectively). This suggested that those transporter moieties, which reached the cell surface, were capable of supporting a normal transport cycle.

**FIGURE 2.**
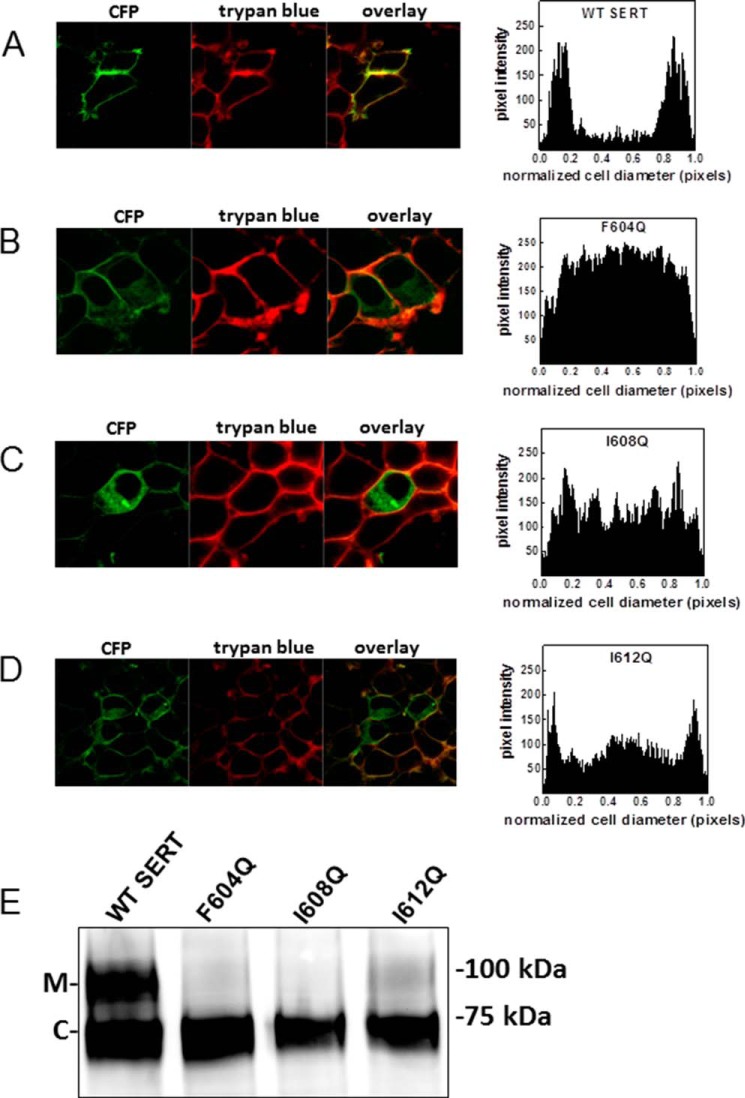
**Mutations in the C-terminal amphipathic helix lead to intracellular retention of SERT.** HEK293 cells were transfected with plasmids encoding CFP-SERT and mutants thereof. After 24 h, the cells were split to perform confocal microscopy and immunoblotting experiments. *A–D*, for confocal microscopy, the transfected cells were seeded onto poly-d-lysine-coated ibidi glass bottom chambers. After 24 h, confocal images were taken, showing CFP-tagged SERT and trypan blue (0.05% in PBS) to visualize the plasma membranes. Overlay images were produced to show colocalization between the signals. The signal distribution (pixel intensity) over the cellular cross-section was determined by ImageJ in at least 10 cells from three or more independent transfections. *E*, membranes were prepared from HEK293 cells transfected with SERT mutants. Samples were separated by SDS-PAGE, blotted onto nitrocellulose, and subjected to immunoblotting using an anti-GFP antibody. The immunoblot is representative of three independent experiments. *C*, core glycosylated; *M*, mature glycosylated.

We also directly verified differences in surface expression by visualizing the cellular distribution of CFP-tagged wild type and mutant versions of SERT: images captured by confocal microscopy showed that wild type SERT was predominantly present at the cell surface (Fig. 2*A*), resulting in extensive colocalization with trypan blue (Fig. 2*A*, *right-hand image*), which was used to delineate the cell membrane. In contrast, CFP-tagged SERT-F604Q (Fig. 2*B*) and SERT-I608Q (Fig. 2*C*) were predominantly retained in the cell. Intracellular retention was also seen with SERT-I612Q, although its surface expression was more readily detected (Fig. 2*D*) than with the other two mutants. This difference in surface expression recapitulates the higher level of substrate uptake and binding (see Fig. 1, *B* and *C*, respectively, *open circles*) with SERT-I612Q than with the other two mutants (Fig. 1, *B* and *C*, *triangles* and *diamonds*). Finally, we resolved cell lysates prepared from cells expressing wild type and mutant versions of SERT by gel electrophoresis to visualize their glycosylation state by immunoblotting: previous experiments verified that the band migrating at 75 kDa was sensitive to enzymatic deglycosylation by endoglycosidase H and thus corresponded to the core glycosylated ER-resident protein (Fig. 2*E*, labeled *C*), whereas the band migrating at 100 kDa (Fig. 2*E*, labeled *M*) was only sensitive to deglycosylation by peptide-*N*-glycosidase F and thus represented the mature glycosylated bands (4). Abundant amounts of the mature form were only seen with wild type SERT (Fig. 2*E*, *left-hand lane*). Some mature glycosylation was also seen in SERT-I612Q (Fig. 2*E*, *right-hand lane*). In contrast, the vast majority of SERT-F604Q and SERT-I608Q were visualized as core glycosylated species (Fig. 2*E*, *second* and *third lanes*). This finding is consistent with the data summarized in Figs. 1 and 2, *A–D*, and confirms that these SERT mutants were retained in the ER.

##### Driving SERT into the Inward Facing Conformation Restores Surface Expression of Helix Mutants

**FIGURE 3.**
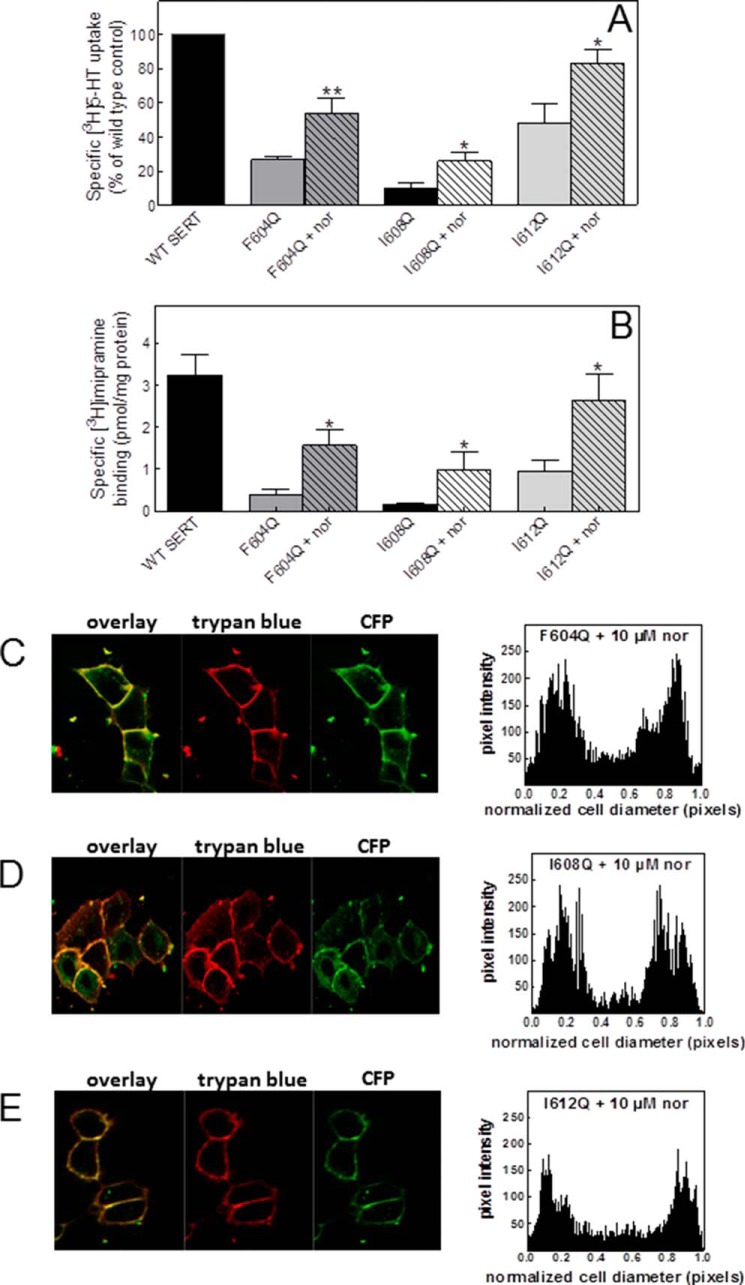
**Noribogaine restores surface expression of helix mutants.** HEK293 cells were transfected with plasmids encoding CFP-SERT and mutants thereof. After 24 h cells were split to perform the assays. *A*, for [^3^H]5-HT uptake, transfected cells were seeded onto 48-well plates and incubated for another 24 h in DMEM containing either 10 μm noribogaine (*nor*) or water (untreated control). Subsequently, cellular uptake of 0.2 μm [^3^H]5-HT was measured in Krebs-HEPES buffer as outlined under “Experimental Procedures.” *B*, membranes (20 μg) prepared from transfected cells were incubated in the presence [^3^H]imipramine (4 nm) as outlined under “Experimental Procedures.” *C–E*, images were captured by confocal microscopy as described under “Experimental Procedures” and in the legend to Fig. 2. The signal distribution (pixel intensity) over the cellular cross-section was determined by ImageJ in at least 10 cells from three independent transfections. *Error bars* in uptake (*A*) and binding experiments (*B*) represent S.E.; Student's paired *t* tests were used to assess the statistical significance of the difference between control and noribogaine-treated cells (*, *p* < 0.05; **, *p* < 0.01).

Previous experiments showed that ER export of SERT-F604Q and SERT-P601A/G602A can be facilitated by pharmacochaperoning these proteins with ibogaine and its demethylated analog noribogaine (5). We explored whether noribogaine also rescued SERT-I608Q and SERT-I612Q. This was the case. When transiently transfected cells were incubated in the presence of 10 μm noribogaine for 24 h and substrate uptake and radioligand binding (Fig. 3*B*) were subsequently determined, this treatment with noribogaine resulted in a significant increase in uptake of [^3^H]5-HT (Fig. 3*A*) and of [^3^H]imipramine binding (Fig. 3*B*). The magnitude of the noribogaine effect was comparable in SERT-I608Q, SERT-I612Q, and SERT-F604Q, which was examined as a reference. The noribogaine-induced increase in surface expression was confirmed by confocal microscopy (Fig. 3, *C–E*). The preferred conformation of noribogaine-bound SERT is the inward facing state (6, 7). Hence, these findings are consistent with the interpretation that driving these SERT mutants into the inward facing state lowers the energy barrier for correct folding and thus promotes ER export of all three helix mutants.

##### The Amphipathic Helix Is Dispensable for ER Export

**FIGURE 4.**
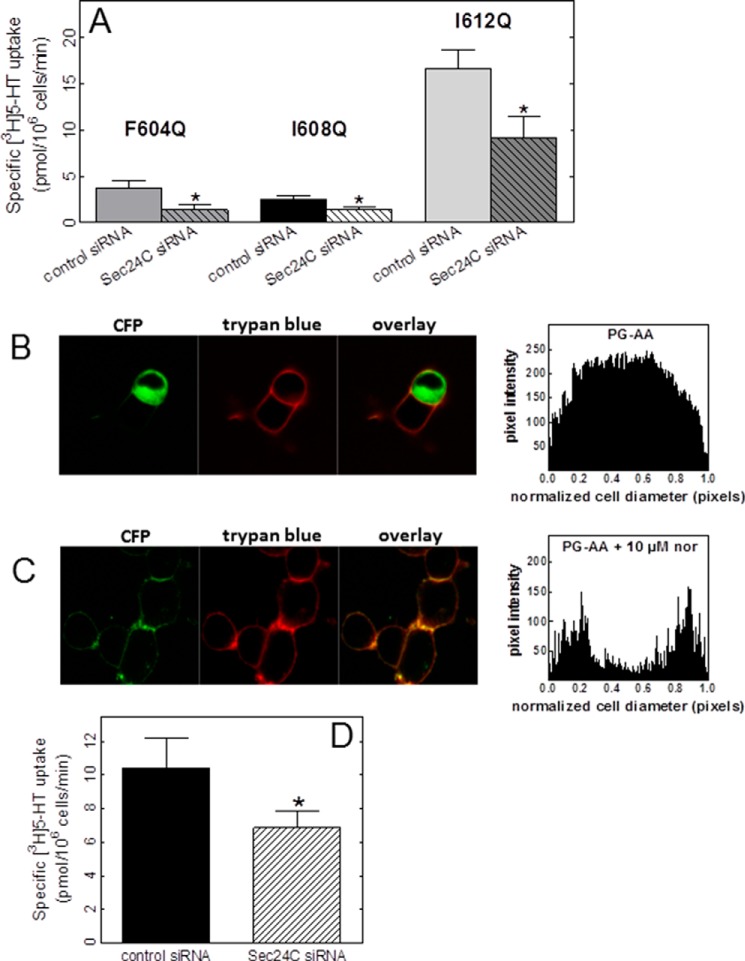
**Surface expression of helix mutants is SEC24C-dependent.** HEK293 cells were transfected with three different siRNAs targeting different portions of the mRNA encoding SEC24C or a mixture of control siRNAs. After 48 h, the cells were transfected with plasmids encoding CFP-SERT mutants. Surface localization of CFP-SERT was measured by [^3^H]5-HT uptake (*A* and *D*) and by confocal microscopy (*B* and *C*) as described in the legends to Figs. 3 and 2, respectively. Where indicated, cells expressing SERT-P601A/G602A (*PG-AA*) were preincubated with 10 μm noribogaine (*nor*) as outlined in the legend to Fig. 3. Experiments were carried out at least three times; *error bars* represent S.E. Student's paired *t* test was used to assess the statistical significance of the difference between control and SEC24C siRNA-transfected cells (*, *p* < 0.05).

ER export of cell surface proteins requires the action of the coatomer protein II complex in which the SEC24 subunit recognizes the client protein as a substrate and recruits it as cargo into the nascent vesicle (32). The C terminus of SLC6 transporters harbors a conserved RI/RL motif, which represents the proposed binding site for SEC24 isoforms (1, 2, 33). In SERT, Arg^607^ and Lys^610^ specify the interaction with SEC24C (1, 2). These residues are within the amphipathic helix. Thus, it is conceivable that mutations in the amphipathic helix abolished SEC24C binding and thus reduced surface expression of the SERT mutants. In this case, residual surface expression of mutant SERT is predicted to be SEC24C-independent (1). As a consequence, depletion of SEC24C should not lead to a further decrease in surface expression of the SERT mutants. We examined this conjecture by siRNA-mediated depletion of SEC24C in HEK293 cells and by subsequently measuring substrate uptake to quantify transporter levels at the cell surface: knockdown of SEC24C uniformly decreased residual uptake mediated by SERT-F604Q, SERT-I608Q, and SERT-I612Q (Fig. 4*A*). This observation indicates that the residual surface levels of these SERT mutants are contingent on SEC24C-dependent ER export. Hence, we conclude that the amphipathic nature of the C-terminal α-helix supports folding, but it is not required for recruiting SEC24C.

We further confirmed this conclusion by examining SERT-P601A/G602A. The residues Pro^601^ and Gly^602^ mark the start of the amphipathic helix (see Fig. 1*A*); their substitution by alanine causes a severe folding defect (4, 5). Accordingly, SERT-P601A/G602A is confined within the cell (Fig. 4*B*), resulting in virtually undetectable substrate uptake (*viz*. Refs. 4 and 7); however, the mutant protein can be effectively pharmacochaperoned by incubating the cells in the presence of noribogaine for 24 h. This pretreatment promoted the delivery of substantial levels of SERT-P601A/G602A to the cell surface (Fig. 4*C*) and allowed for detection of [^3^H]5-HT uptake (Fig. 4*D*). Again, this substrate uptake was significantly decreased if SEC24C was depleted by siRNA-mediated knockdown. At the very least, this observation confirms that noribogaine-triggered surface expression of SERT does not result from an unspecific increase in bulk flow from the ER but still relies on the recruitment of the COPII coat via binding of SEC24C to the C terminus of SERT.

##### Second Site Suppressor Mutations Partially Recapitulate Pharmacochaperoning by Noribogaine

**FIGURE 5.**
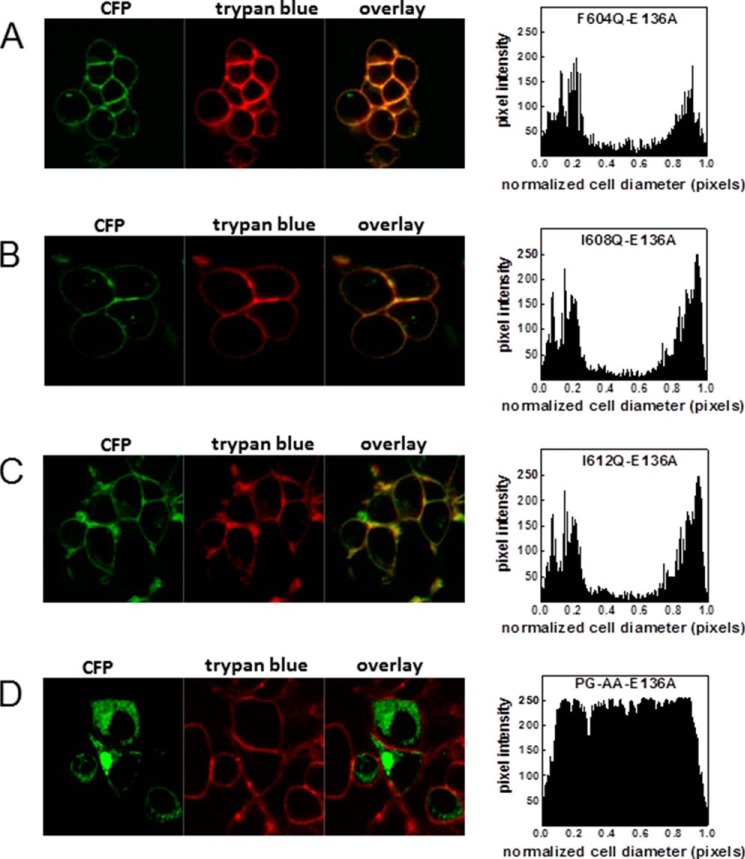
**E136A second site suppressor mutation rescues helix mutants.** The E136A mutation was introduced in addition to the indicated C-terminal mutations (*A–D*). HEK293 cells were transfected with the appropriate plasmids, and confocal microscopy was performed as outlined under “Experimental Procedures” and in the legend to Fig. 2. The signal distribution (pixel intensity) over a cellular cross-section was determined by ImageJ in at least 10 cells from three or more independent transfections. *PG-AA*, P601A/G602A.

Ibogaine and noribogaine drive SERT into the inward facing conformation. Accordingly, mutations that trap SERT in the inward facing state are predicted to promote folding of SERT and thus to compensate for mutations within the amphipathic helix. Substitutions of Glu^136^ of SERT were previously shown to favor the inward facing state of SERT with replacement by alanine showing the most pronounced effect (34). Accordingly, we introduced the E136A mutation into SERT-F604Q, SERT-I608Q, and SERT-I612Q. Uptake of [^3^H]5-HT cannot be used to quantify cell surface levels of SERT-E136A because the protein is trapped in the inward facing state and thus fails to undergo the conformational cycle required for substrate translocation (33). Hence, we examined the cellular distribution of the CFP-tagged double mutants by confocal microscopy (Fig. 5). It is evident that E136A mutation acted as an effective second site suppressor because SERT-E136A/F604Q (Fig. 5*A*), SERT-E136A/I608Q (Fig. 5*B*), and SERT-E136A/I612Q (Fig. 5*C*) were delivered to the cell surface. In contrast, the SERT-P601A/G602A mutation was not rescued by the E136A mutation (Fig. 5*D*).

**FIGURE 6.**
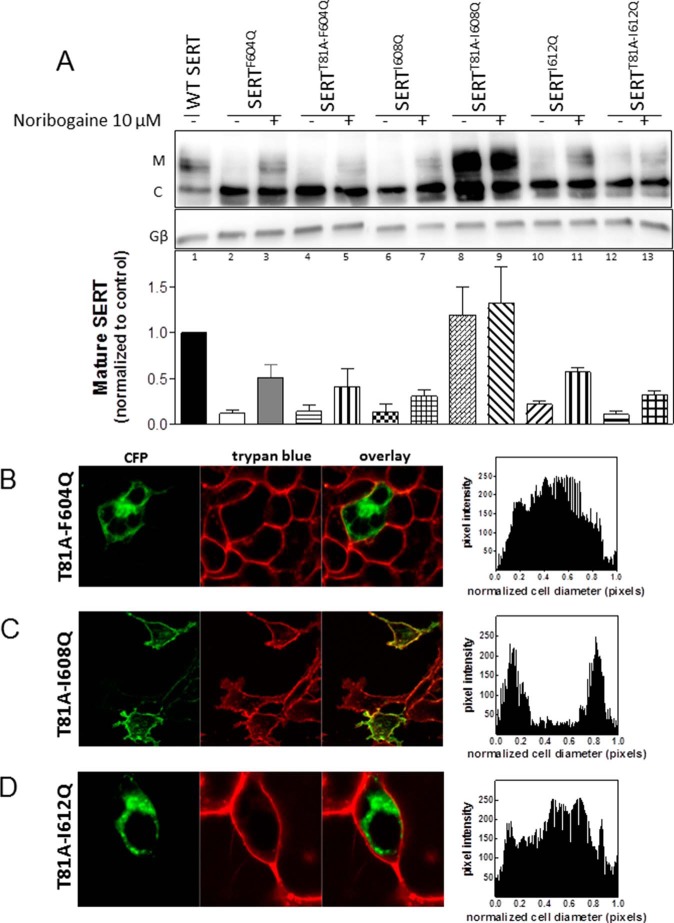
**The T81A mutation drives SERT-I608Q to the cell surface.** The T81A mutation was introduced into SERT constructs harboring the indicated C-terminal mutations. HEK293 cells were transfected with the appropriate plasmids. *A*, detergent lysates (20 μg) were prepared from these cells after 24 h with (+) or without (−) noribogaine (10 μm) treatment, subjected to denaturing gel electrophoresis, transferred onto nitrocellulose membranes, and immunoblotted for the YFP-moiety of SERT (*A*, *upper* blot) and of the G protein β-subunit as a loading control (*A*, *lower blot*). The intensity of the immunoreactive mature glycosylated (*M*) and core glycosylated (*C*) bands from three independent experiments was quantified using ImageJ software. The bar diagram shows the quantification of the mature glycosylated band corrected for the immunoreactivity of Gβ used as a loading control. The level of mature glycosylated form seen in wild type SERT was set to 1, and the levels of the mature glycosylated form in the SERT mutants were expressed as -fold change; *error bars* indicate S.E. *B–D*, images were captured by confocal microscopy as outlined under “Experimental Procedures” and in the legend to Fig. 2. The signal distribution (pixel intensity) over the cellular cross-section was determined by ImageJ in at least 10 cells from three or more independent transfections.

We also introduced the T81A mutation into the helix mutations because this mutation increases the dwell time of SERT in the inward facing conformation (35). Interestingly, T81A only rescued ER export of a single helix mutation, namely SERT-I608Q. This was seen by both immunoblotting (Fig. 6*A*) and confocal microscopy (Fig. 6*C*): there was a substantial increase in the mature glycosylated form of YFP-tagged SERT-T81A/I608Q when compared with SERT-I608Q (Fig. 6*A*, *sixth* and *eighth lanes*). The second site suppressor effect of the T81A mutation exceeded the pharmacochaperoning effect elicited by preincubating the cells with noribogaine (Fig. 6*A*, *seventh* and *eighth lanes*). Conversely, the pretreatment of cells expressing YFP-tagged SERT-T81A/I608Q with noribogaine did not result in any additional increase in the mature glycosylated form (Fig. 6*A*, *eighth* and *ninth lanes*). In contrast, introducing the T81A mutation did not promote the appearance of the mature glycosylated form of SERT-F604Q (Fig. 6*A*, *second* and *fourth lanes*) or of SERT-I612Q (Fig. 6*A*, *10th* and *12th lanes*), although both the single and double mutants were responsive to pharmacochaperoning by noribogaine (Fig. 6*A*, *third*, *fifth*, *11th*, and *13th lanes*). Consistent with these observations, imaging by confocal microscopy visualized CFP-tagged, YFP-tagged SERT-T81A/F604Q (Fig. 6*B*) and SERT-T81A/I612Q (Fig. 6*C*) within the cell.

##### Molecular Modeling Indicates an Ionic Interaction between the C Terminus and IL-1

**FIGURE 7.**
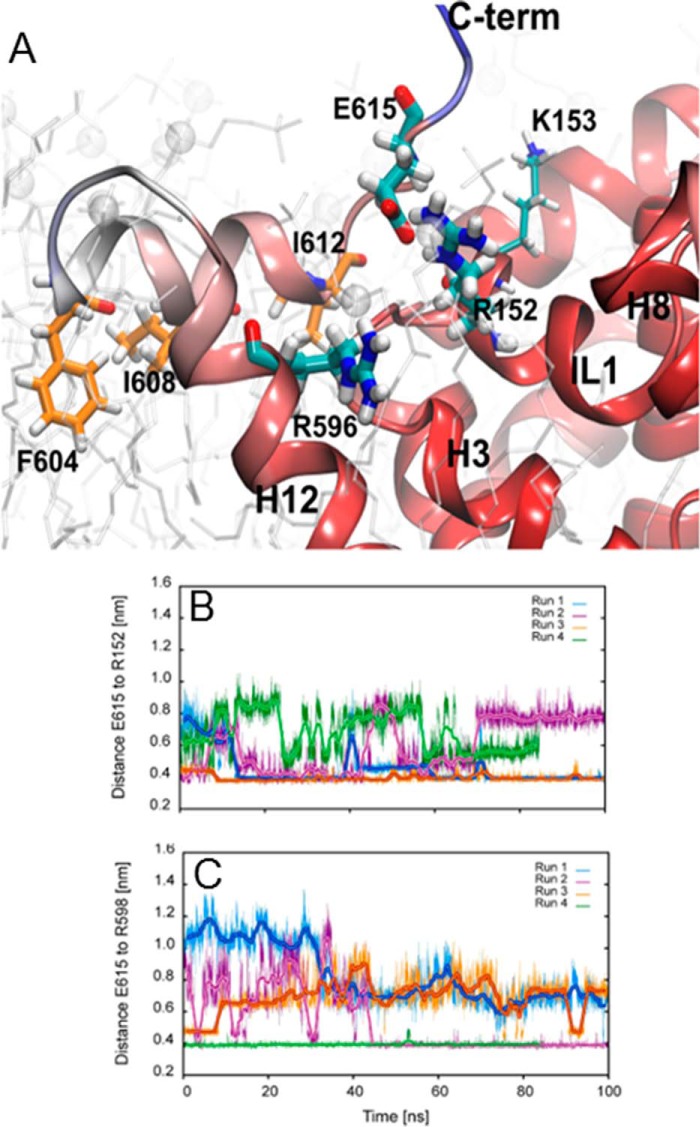
**Simulation of an interaction between the first intracellular loop and the C terminus of SERT.** *A*, snapshot of the cytosolic face focusing on the C terminus of the membrane-inserted and equilibrated structure of SERT. The membrane is indicated in *white* using semitransparent rendering. The backbone of SERT is color-coded by its fluctuations displayed as crystallographic β-factors. The color scale shows low mobility in *red*, intermediate in *white*, and large β-factors in *blue. B*, time evolution of the side chain distance between Glu^615^ and Arg^152^ measured as the distance between Cδ of Glu^615^ and Cζ of Arg^152^ of four independent simulations. We observed the formation of salt bridges in three simulations, two of which were stable. *C*, time evolution of the side chain distance between Glu^615^ and Arg^596^ measured as the distance between Cδ of Glu^615^ and Cζ of Arg^596^ of four independent simulations. The formation of salt bridges was observed in those two simulations that did not result in a stable salt bridge to Arg^152^.

Taken together, the observations indicate that the C-terminal amphipathic α-helix plays a role in the conformational search that results in the stably folded structure. We surmised that this was accomplished by an intramolecular interaction of the C terminus with another segment of SERT. In the crystal structure of dDAT that was recently published (18), there is a cation-π interaction between Trp^597^ in the C terminus of dDAT and Arg^101^ in IL-1. A protein sequence alignment between human SERT and dDAT suggests that Glu^615^ and Lys^153^ of human SERT correspond to Trp^597^ and Arg^101^ of dDAT, respectively. In addition, human SERT carries an arginine at 152 (Arg^152^) as a potential interaction partner for Glu^615^ (Fig. 7*A*). We resorted to a computational approach to understand how the C terminus and IL-1 interact. Four independent simulations of 100-ns length of membrane-inserted SERT with the full-length C terminus were carried out. We observed that residue Glu^615^ interacted dominantly with residue Arg^152^ in intracellular loop 1 (Fig. 7*B*). In addition, the side chain of Glu^615^ was found to form a salt bridge with residue Arg^596^ if not interacting with Arg^152^ (Fig. 7*C*). The time evolution showed that these salt bridges were very stable over time despite exposure to water, charged headgroups of the membrane, and other positively charged residues (Lys^153^, Lys^159^, Lys^605^, Arg^607^, and Lys^610^) in salt bridge-forming distance. A snapshot (Fig. 7*A*) shows the interacting residues Glu^615^ and Arg^152^ on IL-1 and the adjacent residue Arg^596^ in transmembrane helix 12. In the models developed based on the dDAT structure, we found that the side chain of Glu^615^ was very close to residue Arg^152^ and Lys^153^. Interestingly, we observed interactions of Glu^615^ with Arg^152^ in repeated simulations. In contrast, the interaction of Glu^615^ with Lys^153^ was not stable; the ion bridge already broke during the equilibration phase.

In Fig. 7*A*, the backbone of SERT is color-coded for its mobility. Regions with low mobility are shown in *red*, regions with high mobility are in *blue*, and regions with intermediate mobility are shown in *white*. The overall pattern was found to be in line with the crystal structure (18) and the fluctuations observed in simulations of human DAT (17). We observed a stable C-terminal α-helix between residues Thr^603^ and Thr^613^. This helical structure remained stable, but the helix itself showed increased mobility when compared with that of the transmembrane helices. The motion was found to be larger toward Thr^603^. Protein mobility was observed to increase to the level of unrestricted motion only after residue Glu^615^. Interactions of residues at the end of the C-terminal helix with IL-1 apparently stabilized its conformation. The salt bridge interaction of side chain residue Glu^615^ limited the mobility of its backbone, thereby contributing to the structural integrity of the C-terminal helix and its positional stability. We observed in simulations that stabilization was achieved by interactions with either Arg^152^ or Arg^596^. The second set of residues that contribute to the structural stability of the C-terminal helix are residues Phe^604^, Ile^608^, and Ile^612^ on the hydrophobic face of the amphipathic helix. Residues Phe^604^ and Ile^608^ were observed to interact with transmembrane helix 12 and with the hydrophobic core of the membrane. Ile^612^ was found to interact with transmembrane helix 12 and IL-1. Mutation of these residues to the polar residue glutamine would in all three cases disturb the stabilizing interactions. We found that the I608Q mutation was the most detrimental. This can be rationalized as follows: a mutation of Ile^608^ to a polar residue severely disturbs the amphipathic nature of the C-terminal helix because of its central position. In contrast, if mutations are introduced on either end of the helix, two hydrophobic residues are maintained on two consecutive helical turns, thereby preserving the amphipathic nature to some extent.

##### C-terminal Glu^615^ Interacts with Arg^152^ in IL-1 to Promote ER Export

**FIGURE 8.**
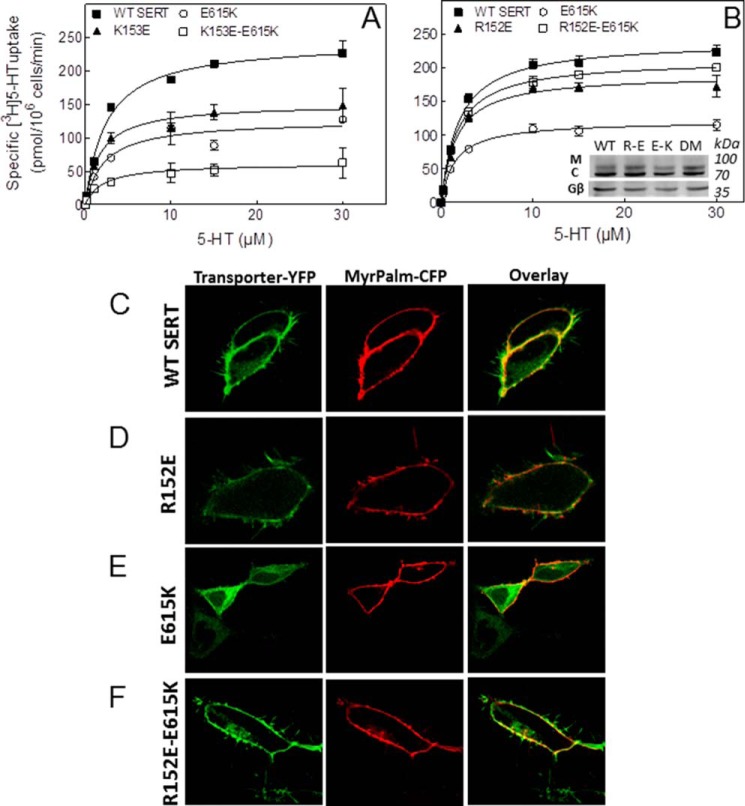
**The first intracellular loop and the C terminus of SERT interact via Glu^615^/Arg^152^.** The indicated single and double mutations (R152E, K153E, and E615K) were introduced into the coding sequence of YFP-SERT, and appropriate plasmids were transiently transfected into HEK293 cells. *A* and *B*, [^3^H]5-HT uptake assays were conducted 48 h after transfection as described under “Experimental Procedures” and Fig. 1. Data are means ± S.E. (*error bars*) from three independent experiments carried out in duplicate. *C–F*, for confocal microscopy, plasmids driving the expression of YFP-tagged wild SERT (*C*), SERT-R152E (*D*), SERT-E615K (*E*), and SERT-R152E/E615K (*F*) were transiently cotransfected (at a ratio of 4:1) with a plasmid encoding myristoylated and palmitoylated CFP (*MyrPalm-CFP*) as a surface marker. Confocal microscopy was performed as outlined under “Experimental Procedures.” *M*, mature glycosylated; *C*, core glycosylated; *DM*, double mutant R152E/E615K; *R-E*, R152E; *E-K*, E615K.

The most important insight of the simulations was the salt bridge between the end of the C-terminal α-helix and IL-1 of SERT. We verified its relevance by introducing point mutations to create SERT-E615K, SERT-K153E, and SERT-R152E. The rationale for this approach was to assume that the single mutations ought to reduce surface expression by disturbing the ionic interaction between the C terminus and IL-1. This was the case: uptake of substrate by SERT-E615K for instance was reduced by 50% (Fig. 8, *A* and *B*, *open circles*). Similarly, the maximum velocity of substrate translocation by SERT-K153E was also decreased (Fig. 8*A*, *closed triangles*). Surprisingly, the phenotypic consequence of the SERT-R152E mutation was modest (Fig. 8*B*, *closed triangles*). However, if the mutations were combined, *i.e.* the charges in the first intracellular loop and at the end of the C-terminal α-helix were reversed, the results were unequivocal: the K153E mutation failed to rescue the E615K mutation; in fact, SERT-K153E/E615K (Fig. 8*A*, *open squares*) was less active than either single point mutant. In contrast, the R152E mutation counteracted the effect of mutating Glu^615^ to Lys such that the transport rate of SERT-R152E/E615K (Fig. 8*B*, *open squares*, and Table 1) approached that of wild type SERT (Fig. 8*B*, *closed squares*, and Table 1). This was also recapitulated if the ratio of mature and core glycosylated SERT was visualized by immunoblotting: the mature glycosylated band was less abundant in SERT-E615K (Fig. 3, *inset*, *lane* labeled *E-K*); however, it was restored to wild type levels in the double mutant SERT-R152E/E615K (Fig. 8*B*, *inset*, *lane* labeled *DM*). Finally, we also verified the cellular distribution of CFP-tagged SERT-E615K and SERT-R152E/E615K by confocal microscopy. It is evident that a substantial amount of SERT-E615K was retained within the cells (Fig. 8*E*) because a large portion of the YFP fluorescence did not co-localize with the fluorescently labeled plasma membrane (Fig. 8*E*, *left-hand image*). In contrast, in cells expressing SERT-R152E/E615K, the bulk of the YFP fluorescence co-localized with the fluorescence of myristoylated and palmitoylated CFP (Fig. 8*F*, *left-hand image*). Consistent with the uptake data, the ratio of mature to core glycosylated band of the single mutant SERT-R152E (Fig. 8*B*, *inset*, *lane* labeled *R-E*) was comparable with that of wild type SERT (Fig. 8*B*, *inset*, *lane* labeled *WT*); the cellular distribution also showed that SERT-R152E reached the cell surface (Fig. 8*D*) to the same extent as wild type SERT (Fig. 8*C*).

**TABLE 1:** **Kinetic parameters for substrate uptake by SERT mutants affecting the ionic interaction between the first intracellular loop and the C terminus** HEK293 cells were transfected with plasmids driving the expression of wild type and mutant versions of SERT affecting the putative ionic interaction between the C terminus and the first intracellular loop. After 48 h, uptake of [^3^H]5-HT was measured as described under “Experimental Procedures.” The *K_m_* and *V*_max_ values are shown as arithmetic means ± S.E. from three independent experiments performed in triplicate and shown in Fig. 8. Differences were compared for statistical significance by repeated measures analysis of variance followed by Tukey's post hoc *t* tests.

	[^3^H]5-HT uptake	
	*K_m_*	*V*_max_
	μ*m*	*pmol/10^6^ cells/min*
Wild type SERT	1.8 ± 0.2	182 ± 9
SERT-R152E	1.7 ± 0.4	143 ± 10
SERT-E615K	1.4 ± 0.1	89 ± 11*^a^*
SERT-R152E/E615K	2.0 ± 0.4	167 ± 3

*^a^ p* < 0.001, significantly different from all other SERT variants.

**FIGURE 9.**
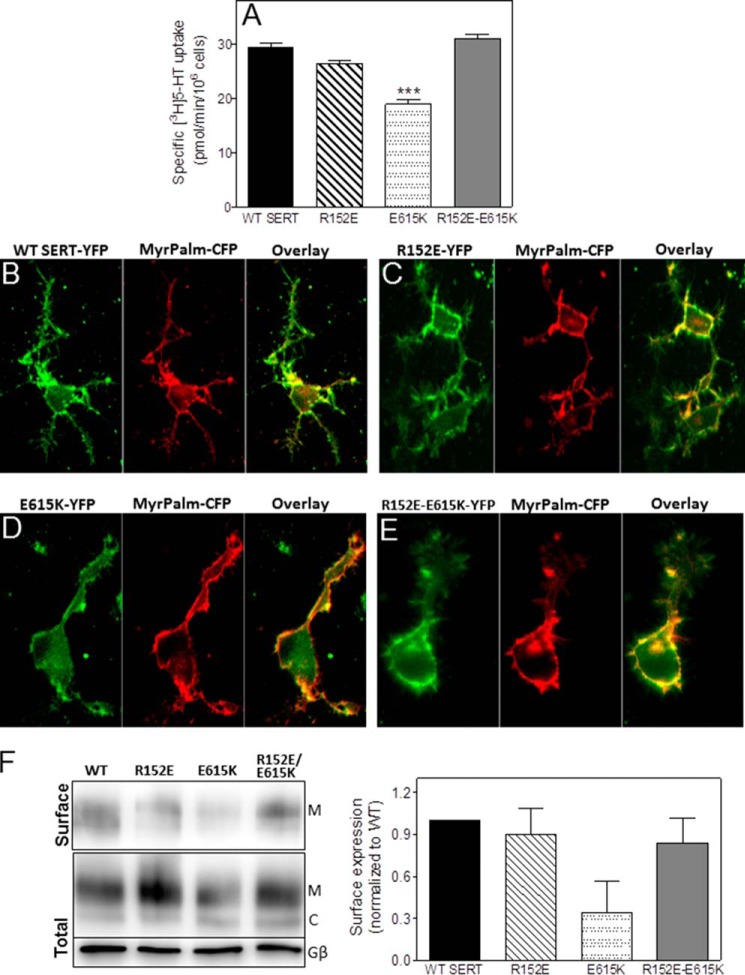
**The effects of mutating residues Glu^615^ and Arg^152^ are recapitulated in the neuronal CAD cell line.** Plasmids encoding wild type SERT and single and double mutations (R152E, K153E, and E615K) were transiently transfected into CAD cells. *A*, [^3^H]5-HT uptake was measured 48 h after transfection as described under “Experimental Procedures.” Data are means ± S.E. (*error bars*) from three independent experiments carried out in triplicate. One-way analysis of variance followed by Tukey's post hoc *t* test was used to examine the statistical significance of the difference between cells expressing the wild type or mutant SERTs (***, *p* < 0.001). *B–E*, confocal microscopy images of differentiated CAD cells expressing the indicated YFP-tagged wild SERT (*B*), SERT-R152E (*C*), SERT-E615K (*D*), and SERT-R152E/E615K (*E*) transiently cotransfected (at a ratio of 4:1) with a plasmid encoding myristoylated and palmitoylated CFP (*MyrPalm-CFP*) to mark the plasma membrane. CAD cell differentiation was induced by serum removal from the culture medium 24 h prior to confocal microscopy. *F*, biotinylation of cell surface proteins was carried out in CAD cells expressing the indicated versions of SERT. *Top panel*, surface expression; *bottom panels*, total lysate fractions and G protein β-subunit (as a loading control). The integrated intensity of the biotinylated bands (*M*) was quantified by ImageJ relative to the total amount of SERT immunoreactivity in the lysate (*i.e.* the bands with mature glycosylation (*M*) and core glycosylation (*C*)) and normalized to wild type. The data are means ± S.E. (*error bars*) (*n* = 4).

We also verified these observations in a cell line of neuronal origin: CAD cells are derived from neuronal tumor cells arising in transgenic mice in which the SV40 large T antigen was placed under the control of the tyrosine hydroxylase promoter. CAD cells express neuron-specific proteins, such as class III β-tubulin, GAP-43, SNAP-25, and synaptotagmin; they can be differentiated upon serum removal from the culturing medium such that they sprout neurite extensions (36). Most importantly, they endogenously express the norepinephrine transporter and hence have the specialized machinery to fold and sort SLC6 transporters, *e.g.* GAT1 and SERT (1, 37). (i) As in HEK293 cells, expression of SERT-E615K in CAD cells resulted in lower substrate uptake (Fig. 9*A*, *third bar*). Expression of the double mutants restored uptake velocity to that seen in CAD cells expressing wild type (Fig. 9*A*, *fourth* and *first bars*). (ii) SERT-E615K showed substantial intracellular retention in differentiated CAD cells (Fig. 9*D*) compared with wild type SERT (Fig. 9*B*), SERT-R152E (Fig. 9*C*), and the rescued SERT-R152E/E615K double mutant (Fig. 9*E*). (iii) We also visualized the levels of transporters at the plasma membrane by biotinylation of cell surface proteins: when compared with wild type SERT, SERT-E615K was present at lower amounts at the cell surface (Fig. 9*F*, *first* and *third lanes*). The second site suppressor restored cell surface levels such that the amount of biotinylated SERT-R152E/E615K was comparable with that of wild type SERT (Fig. 9*F*, *fourth* and *first lanes*).

## Discussion

Folding of a protein into its final and correct conformation is a search process during which a protein is thought to adopt a multitude of different alternative conformations. This presumably also applies to folding of proteins with several membrane-spanning segments where the conformational search space is restricted by their lateral mobility in the membrane. The folding trajectory can be pictured as a movement along a funnel-like energy landscape with local minima (38, 39). The local minima are populated by conformational states, which engage chaperone proteins located in both the ER lumen (40, 41) and the cytosol (38). Our previous experiments show that the C terminus of SERT engages HSP70-1A and HSP90β (5). This suggests that the C terminus is involved in the conformational search, which leads to the stably folded state of SERT. The following observations are in line with this hypothesis. (i) The segment of the C terminus from Phe^604^ to Thr^613^ forms an amphipathic α-helix. If the amphipathic nature was disrupted by replacing the hydrophobic side chains of F^604^, I^608^, or I^612^ by the hydrophilic residue glutamine, folding of the resulting SERT mutants was severely impaired such that they were retained in the ER in a state that was not capable of binding the cognate SERT ligand imipramine. (ii) This folding defect was remedied in part by pharmacochaperoning with noribogaine or by second site suppressor mutations. In either case, surface expression of these mutants was restored by stabilizing the inward facing conformation of SERT. (iii) Molecular dynamics simulation identified an intramolecular interaction that linked the C terminus to the first intracellular loop of SERT via an ionic interaction of Glu^615^ with Arg^152^. The role of this bond in folding was confirmed by rescuing surface expression of the charge reversal, double mutant SERT-R152E/E615K. It should be noted that our simulations start from the human SERT modeled onto the outward facing *D. melanogaster* DAT (18). We argue here and in our earlier work (4) that the folding trajectory moves through the inward facing conformation. The outward facing structure of the dDAT is the only crystal structure of monoamine transporters available to date and the only one that includes the C-terminal helix. However, the local structure within the investigated region (transmembrane helix 12 and IL-1) is not expected to change between the inward and the outward facing states. This inference is based on a comparison of the inward facing (42) and the outward facing LeuT structures (43), which show essentially identical positions of IL-1 and transmembrane helix 12 after a structural superposition of the scaffold domain.

This interaction recapitulates the intramolecular interaction, which was visualized in the crystal structure of *Drosophila* DAT: a cation-π interaction is formed between the guanidino group Arg^101^ in the first intracellular loop and the aromatic ring of Trp^597^, which similar to Glu^615^ in human SERT is located two amino acids downstream from the end of the C-terminal α-helix (18). (iv) Although the sequence conservation in C-terminal α-helix human SERT and *Drosophila* DAT is modest, both share another key structural feature. In both proteins, the α-helix is flanked by prolylglycine (^601^PG^602^ in SERT) at the N-terminal start and by a proline residue on the C-terminal end. The importance of ^601^PG^602^ for folding of SERT was already appreciated previously (4, 5) and was again highlighted in the present study by the observation that the folding defect of SERT-P601A/G602A was not rescued by introducing the E136A second site suppressor mutation. Replacement of ^601^PG^602^ is predicted to destroy the tight turn, which positions the C-terminal α-helix. Hence, these findings are again consistent with the interpretation that the C-terminal α-helix participates in the conformational search, which results in the folded state of SERT.

Although our observations showed that the ionic interaction between Glu^615^ and Arg^152^ facilitates folding of SERT, we stress that this interaction is not indispensable: in fact, the folded state can be reached in its absence. This is exemplified by the fact that surface levels of SERT-E615K still reached about 50% of wild type SERT levels. Similarly, Arg^152^ can be mutated without phenotypic consequence. The difference between SERT-E615K and SERT-R152K can be rationalized by considering the insights obtained by molecular dynamics simulations that revealed a substantial conformational flexibility in the C terminus of SERT: the C-terminal helix Glu^615^ can be stabilized by interacting either with Arg^152^ or Arg^596^. In SERT-R152K, the interaction with Arg^596^ is preserved, whereas in SERT-E615K, neither interaction is possible. Thus, in the absence of any stabilizing interaction, the energy barrier, which has to be overcome in the folding trajectory, is raised, resulting in impaired folding and reduced surface expression. This model also allows rationalization of earlier truncation experiments: deletion of the last 15 C-terminal residues does not impair folding and surface delivery of the resulting SERT-Δ15 to any appreciable extent (4, 44). This indicates that the very C terminus does not participate in the conformational search and confirms the importance of Glu^615^, which is the last residue in SERT-Δ15. Surface delivery of SERT-Δ16 is reduced, whereas SERT-Δ17 is retained in the ER in an inactive, misfolded state (4, 44). The last residues in SERT-Δ16 and SERT-Δ17 are Pro^614^ and Thr^613^, respectively. Although the truncation experiments imply an important role of Pro^614^, its replacement by alanine does not impair folding or surface delivery of full-length SERT or SERT-Δ16 (4, 44). Thus, it is the length of the SERT C terminus that is relevant for SERT folding rather than the presence of Pro^614^. The partial phenotype of SERT-Δ16 can be accounted for by the free carboxylate group provided by Pro^614^, which may substitute for that otherwise provided by the side chain of Glu^615^ and thus still support some interaction with Arg^152^. We note that, in contrast to SERT-R152E, surface delivery of SERT-K153E was reduced by about 50%. At present, we are at a loss to explain the phenotype of this mutant, but we consider it unlikely that Lys^153^ provides an alternative counter-ion in the first intracellular loop for Glu^615^ because the charge reversal, double mutant resulting in SERT-K153E/E615E further compromised surface delivery.

The conformational flexibility of the C terminus, which was highlighted by the molecular dynamics simulations, is consistent with the conjecture that the C terminus of SERT may adopt several conformations to allow for recruitment of different interaction partners (29). In fact, it is worth noting that, provided they were pharmacochaperoned into a stably folded conformation, all SERT mutants examined still recruited SEC24C despite the disruption of the amphipathic nature of the C-terminal α-helix (in SERT-F604Q, SERT-I608Q, and SERT-I612Q) or despite the distortion introduced by eliminating the turn preceding the α-helix (in SERT-P601A/G602A). It is also evident from an inspection of Fig. 7*A* that the conformation depicted therein is unlikely to be the one selected by SEC24C: the residues, which specify interaction of SERT with SEC24C and of DAT, norepinephrine transporter, and GAT1 with SEC24D (1), are not readily accessible. Similarly, the conformational flexibility of the C terminus may also be important to allow for visiting alternative local minima in the folding trajectory. All mutants examined, including SERT-P601A/G602A, were pharmacochaperoned by noribogaine. Thus, binding of noribogaine effectively lowered the energy barrier to allow the proteins to escape from their stalled intermediate conformational states and proceed along the folding trajectory. In contrast, the remedial action of the E136A mutation was restricted to those mutations that disrupted the amphipathic nature of the C-terminal α-helix. This is consistent with our earlier observation that SERT-P601A/G602A and SERT-F604A are stalled at different stages along the folding trajectory (5). Finally, the T81A mutation only rescued SERT-I608Q and achieved this remarkable efficacy. This finding is striking because this mutant has the most severe folding defect among the helix mutations. This leads to the conclusion that, although only separated by a single helical turn from Phe^604^ or Ile^612^, Ile^608^ has a distinctive contribution to folding: its substitution by a hydrophilic residue traps the transporter at a local energy minimum in the folding trajectory, which is visited neither by SERT-F604Q nor by SERT-I612Q. Accordingly, the conformation favored by the T81A mutation only allows the conformational search to proceed from the position at which SERT is otherwise confined by the I608Q mutation.

There are more than 50 mutations of transporter proteins of the SLC6 class that give rise to clinically relevant phenotypes (8). Several of these are associated with misfolding of the transporter, resulting in folding diseases. Thus, understanding the folding trajectory is important to develop mechanism-based approaches to remedy these folding diseases (8). In addition, the current insights may be relevant to other membrane proteins. In fact, G protein-couples receptors also have a C-terminal amphipathic α-helix (helix 8), which runs perpendicular to the transmembrane helices and parallel to the plane of the membrane. This α-helix likely participates in the conformational search during the folding trajectory because the folding state of the receptor is also sampled by a heat shock protein relay (45) and because mutations within the C-terminal helix result in misfolding (46, 47) that can be remedied by pharmacochaperoning (47, 48). The analogies to the current observations are obvious. We suspect that this will also extend to other classes of membrane proteins with intracellular C termini.

## Footnotes

4 C. Neale and R. Pomès, personal communication.

## Footnotes

3 The abbreviations used are:

SERT: serotonin transporter
5-HT: 5-hydroxytryptamine (serotonin)
CFP: cyan fluorescent protein
COPII: coatomer protein complex II
DAT: dopamine transporter
dDAT: *D. melanogaster* DAT
ER: endoplasmic reticulum
HSP: heat shock protein
IL-1: first intracellular loop
SLC6: solute carrier 6.
